# The complete mitochondrial genome analysis of *Elachiptera decipiens* (Loew, 1863) (Diptera: Chloropidae)

**DOI:** 10.1080/23802359.2026.2652762

**Published:** 2026-04-10

**Authors:** Yong-Yue Gu, Yue Guo, Wan-Long Zhang, Heng-Jia Zhang, Ai-Mei Dai, Hao Zhang

**Affiliations:** ^a^ College of Plant Protection, Key Laboratory of Integrated Pest Management on Crops in Northwestern Loess Plateau of Ministry of Agriculture and Rural Affairs, Northwest A&F University, Xianyang, China; ^b^ Agricultural Technology Promotion Center of Bole Mongol Autonomous Prefecture, Bole, China

**Keywords:** Mitogenome, Chloropidae, Oscinellinae, *Elachiptera decipiens*

## Abstract

*Elachiptera decipiens* is cereal pest distributed in North America, Europe, and China. Its complete mitochondrial genome from Xinjiang, China was sequenced using next-generation sequencing technique. The 18,344 bp mitogenome contains 13 protein-coding gene sequences (PCGs), 22 tRNAs, and 2 rRNAs. Phylogenetic analysis based on 13 PCG sequences at family level and *COI* gene sequences at subfamily level. Phylogenetic analysis revealed that *E. decipiens* is closely related to *E. insignis*. The complete mitogenome of *E. decipiens* would help understand Chloropidae evolution and develop rapid identification methods based on mitochondrial genes.

## Introduction

*Elachiptera decipiens* (Loew, 1863) (Diptera: Chloropidae: Oscinellinae) is mainly distributed in Canada, the United States, and some countries in Europe. In 2021, it was first discovered in Bole City of Xinjiang Uygur Autonomous Region, China, and since then it has become an important pest on common wheat (*Triticum aestivum* L.). The larva of *E. decipiens* feeds within the wheat stem, leading to stunting, distortion, and decreased tillering. The most evident symptom is the manifestation of the ‘white head’ condition. In this condition, the seed head undergoes necrosis and turns white, whereas the lower stem areas and leaves retain their green coloration. However, due to the strikingly similar morphological features and symptoms caused by the larvae, it is significantly challenging to identify *E. decipiens* from other Chloropids species in wheat fields, such as *Meromyza saltatrix* (L.) and *Oscinella pusilla* (Meigen) (Liu and Sun 1965; Wang and Yang 1990). *E. decipiens* also has the ability to damage maize (*Zea mays* L.) (Federal Research Centre for Cultivated Plants 2021).

Despite the fact that this pest has been widely distributed in North America and Europe for a long time, the studies on the biology, ecology, and genetics are still limited. In the present study, we sequenced the complete mitochondrial genome of *E. decipiens* for the first time and conducted the phylogenetic analysis.

## Materials and methods

The larvae and pupae of *E. decipiens* were collected from Bole city (E 82.0046°, N 44.9255°), Xinjiang Uyghur Autonomous Region, China, in July 2021. All samples were live-collected and initially preserved in 5 ml centrifuge tubes in fields, and then were transferred to laboratory for rearing to adulthood (collected by Aimei Dai and Wanlong Zhang). The adult specimens have been deposited in the Insect Museum of College of Plant Protection, Northwest A&F University, and the certificate number is DI001 (contact person: Hao Zhang, email: zhh1972@nwsuaf.edu.cn).

The specimen was identified morphologically by Yue Guo by referencing Mlynarek and Wheeler (2018), and DNA sequencing. DNA samples were pooled using next-generation library construction following Gillett et al. (2014) on the Illumina NovaSeq 6000 platform by Personal Biotechnology Co., Ltd. (Shanghai, China). The raw reads were filtered and trimmed using Fastp (https://github.com/OpenGene/fastp) and Adapter Removal (version 2) following Schubert et al. (2016). *De novo* assemblies of high-quality reads were conducted using A5 miseq v20150522 (Coil et al. 2015) and SPAdes v3.9.0 (Bankevich et al. 2012). Following the completion of genome assembly using Bandage software (v0.8.1, https://github.com/rrwick/Bandage), a visualization analysis was conducted on the GFA format generated from the map file to confirm that the genomic DNA fragments formed complete circular molecules. Based on the connectivity and sequence information of each node in the graph, the complete genome sequence file was reconstructed through manual proofreading. Re-BLAST analysis revealed that sequencing depth was evenly distributed across most regions of the genome, thereby validating the completeness and accuracy of the assembly results. However, a reduction in sequencing depth was observed in AT-rich regions of the genome, likely due to amplification bias during PCR and bridge amplification, leading to decreased coverage in these areas. The gene annotation was performed using Mitos WebServer (http://mitos.bioinf.uni-leipzig.de/index.py) (Bernt et al. 2013), and the determination of the control region boundary was determined using the automatic annotation function of MitoS2. The circular map of the mitogenome was drawn using CGView Visualization software (Alikhan et al. 2011).

To determine the taxonomic position of *E. decipiens*, analyze the phylogenetic analysis was carried out using 13 protein-coding gene (PCG) sequences from 11 Chloropidae taxa, including *E. decipiens*, at family level with *Mayetiola destructor* (Say) (Diptera: Cecidomyiidae) and *Drosophila busckii* Coquillett (Diptera: Drosophilidae) as outgroups. Meanwhile, given the critical role of the *COI* gene in insect phylogeny and evolution, *COI* gene sequences from 10 species from Oscinellinae taxa were used for phylogenetic analysis at subfamily level with the two closely related species from subfamily Chloropinae, *Meromyza saltatrix* (L.) and *Cetema elongatum* (Meigen) as outgroup. All the 13 PCGs and COI gene sequences were downloaded from GenBank. All the sequences were concatenated and aligned in DNAman 6.0. The maximum-likelihood (ML) method was employed to analyze the phylogenetic tree. The ML analysis was performed by MEGA 11 with 1000 bootstrap replicates.

## Results

**Figure 1.**
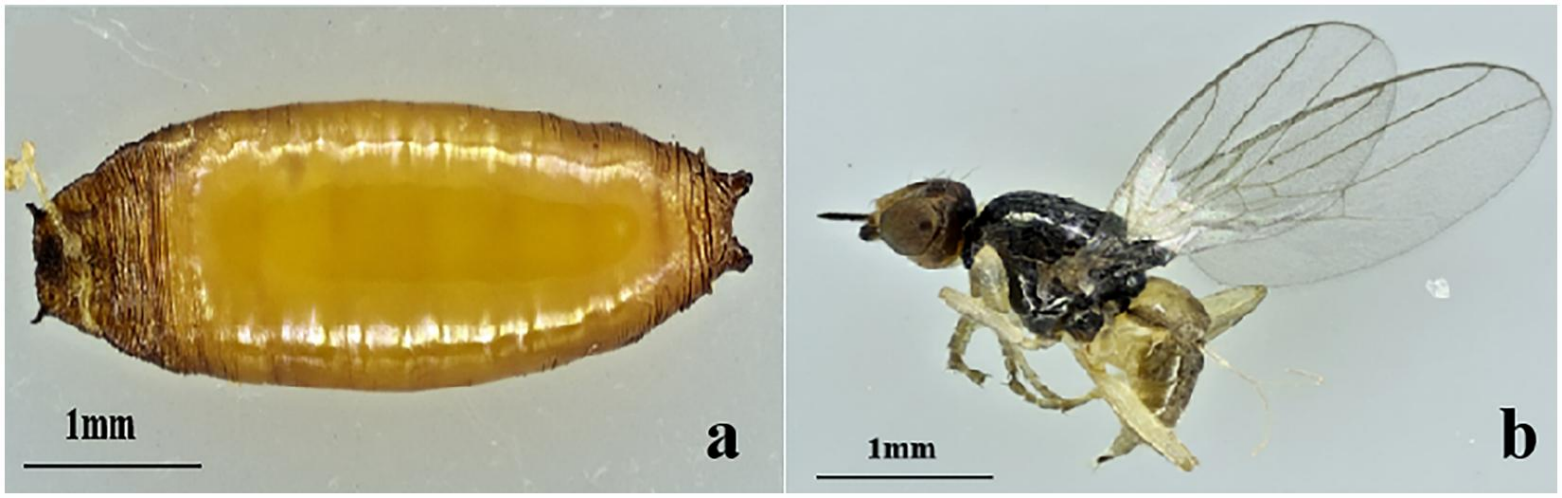
*Elachiptera decipiens* larva and adult. (a) Larva; (b) adult. Photos were taken and edited by Yue Guo.

**Figure 2.**
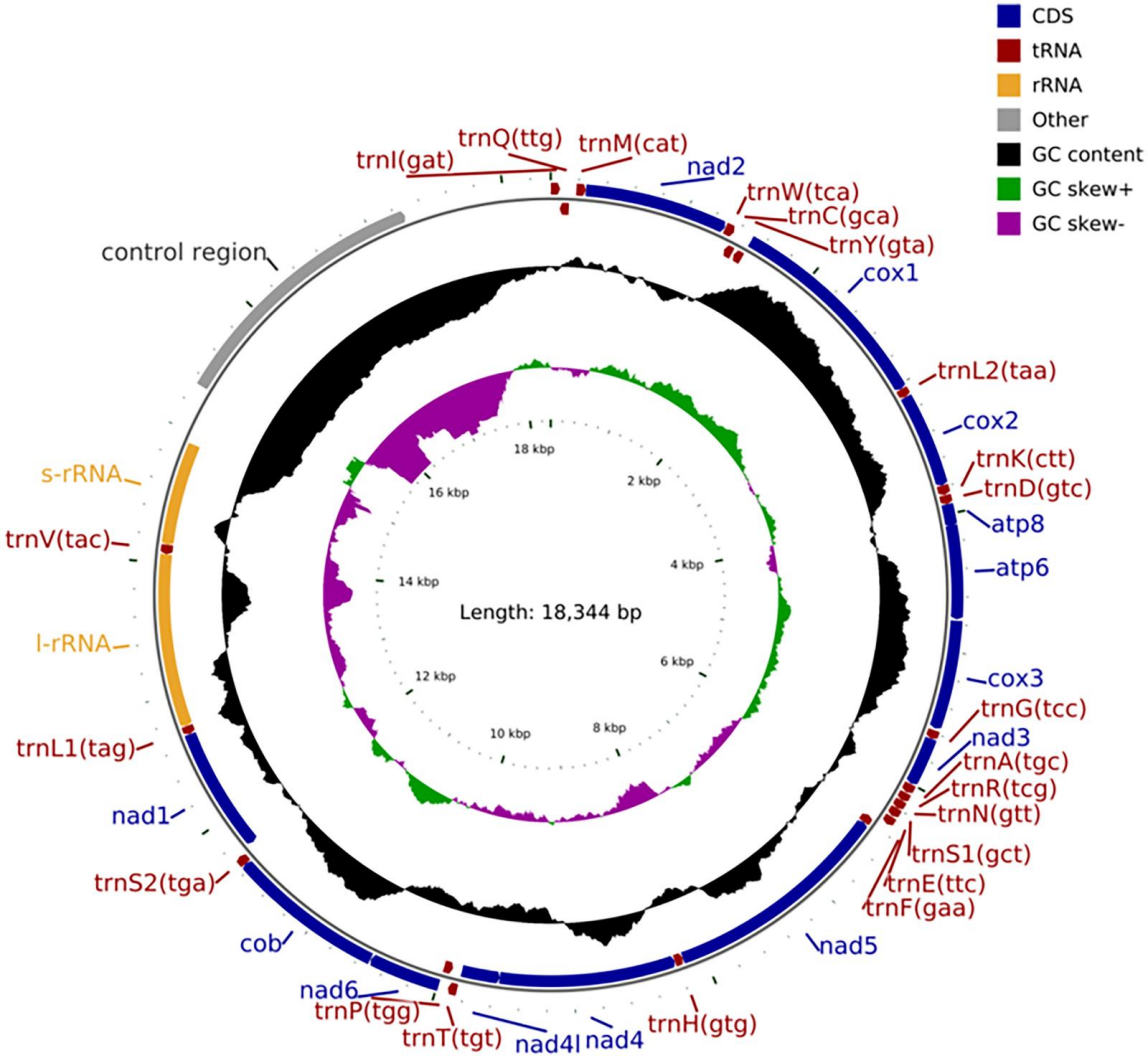
Mitochondrial genome map of *Elachiptera decipiens*. From inside to outside, the first circle represents the scale; the second circle represents GC skew; the third circle represents GC content; the fourth and fifth circles represent the arrangement of protein coding, tRNA, and rRNA genes on the genome.

The mitochondrial genome of *E. decipiens* (Figure 1), was assembled as a circular molecule measuring 18,344 bp (GenBank: PQ585796). It exhibited sequencing coverage depths varying from 59× to 1074×, with an average depth of 773.388× (Figure S1). The overall nucleotide composition was notably AT-rich, with A, T, C, and G contents of 42.89%, 39.59%, 10.10%, and 7.42%, respectively, showing in a total AT bias at 82.48%. This genome contained 13 PCGs, 22 transfer RNA genes, two ribosomal RNA genes (s-rRNA and l-rRNA), and a control region (A–T rich region/D-loop) (Figure 2). The combined length of all PCGs was 11,218 bp, representing 61.2% of the entire mitochondrial genome. The majority of PCGs started with standard ATN initiation codons, except for cox1 (TCG), nad1 (TTG), and nad5 (GTG). In terms of termination, eight PCGs ended with a complete TAA stop codon, while cob ended with TAG, and four genes—cox1, cox2, nad4, and nad5—used a single T as an incomplete stop codon. Gene overlaps were observed in 11 regions, totaling 21 bp, with the longest overlap (8 bp) occurring between trnW and trnC. Additionally, intergenic spacers spanned a total of 562 bp, ranging from 1 bp to 1070 bp. The longest spacer, measuring 1070 bp, was located between trnI and the D-loop region.

**Figure 3.**
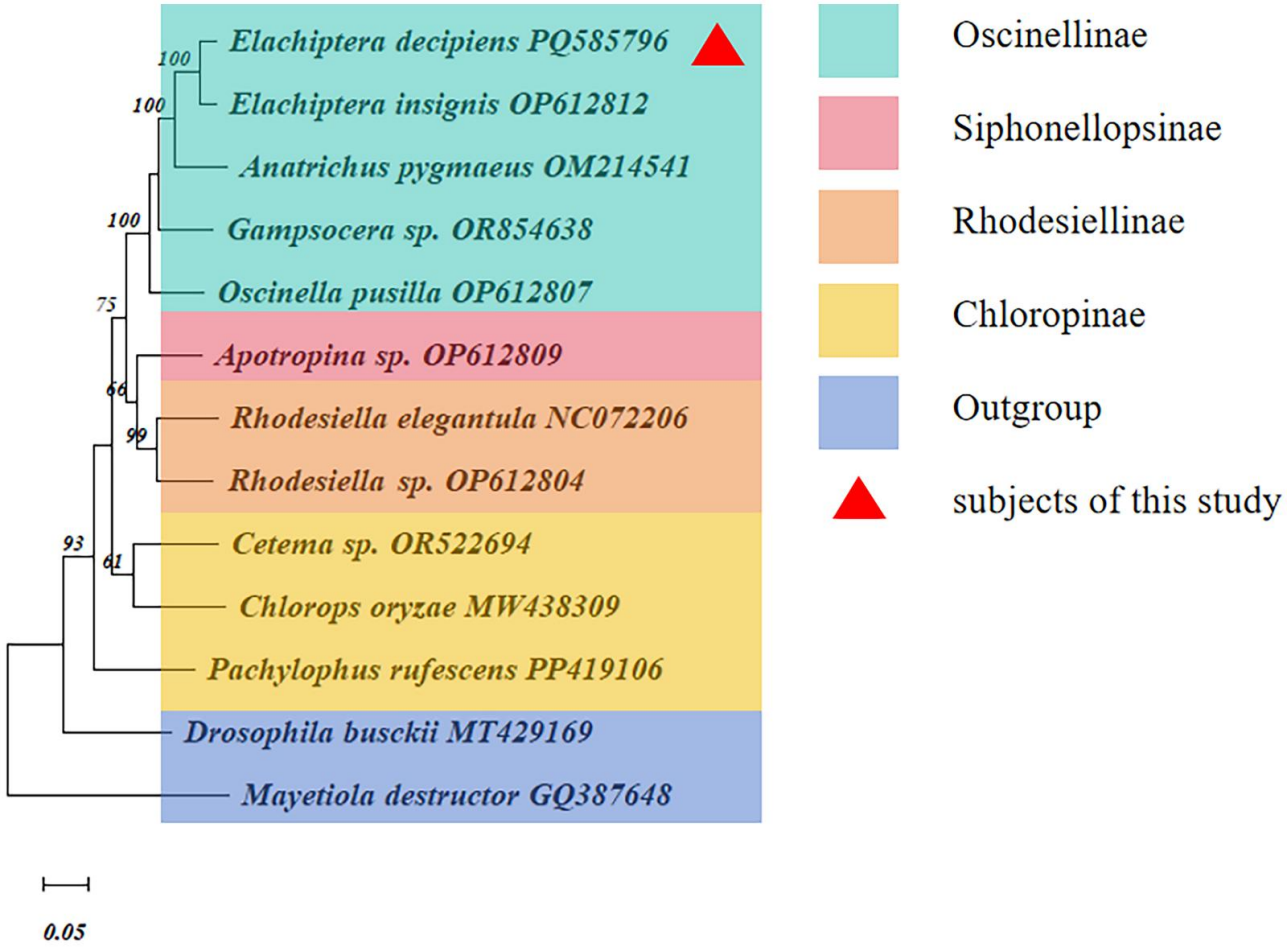
ML phylogenetic tree based on the 13 protein-coding genes (PCGs). The larger the number on the branch, the higher the proof reliability. Alphanumeric terms indicate the accession number of the GenBank. The following sequences were used: *Elachiptera decipiens* PQ585796 (this study); *Elachiptera insignis* OP612812 (Liu et al. 2024); *Anatrichus pygmaeus* OM214541 (Cai et al. 2022); *Gampsocera* sp. OR854638 (He et al. 2024); *Oscinella pusilla* OP612807 (Liu et al. 2024); *Apotropina* sp. OP612809 (Liu et al. 2024); *Rhodesiella elegantula* NC072206 (Liu et al. 2024); *Rhodesiella* sp. OP612804 (Liu et al. 2024); *Cetema* sp. OR522694 (Liu et al. 2024); *Chlorops oryzae* MW438309 (Wang et al. 2021); *Pachylophus rufescens* PP419106 (Liu et al. 2024); *Drosophila busckii* MT429169 (Zhang and Jin 2020); *Mayetiola destructor* GQ387648 (Beckenbach and Joy 2009).

The phylogenetic tree of Chloropidae in this study based on 13 PCGs showed that the newly sequenced species *E. decipiens* was clustered in the subfamily Oscinellinae clade and had the closest relationships with *E. insignis* NC072208, but had a distant relationship with other species belonging to subfamily Siphonellopsinae, Rhodesiellinae, and Chloropinae (Figure 3). Based on *COI* gene sequences, the phylogenetic tree showed that the studied species was clustered together with *Elachiptera decipiens* JF874163 with a high bootstrap support values (BSV = 100), indicating that it is *E. decipiens*. Furthermore, *E. decipiens* was closely associated with *E. insignis* NC 072208 and *E. cornuta* HE614017, then with *Anatrichus pygmaeus* and *Melanochaeta pubescens*, but had a distant relationship with *Oscinella pusilla*, *Cadrema minor*, and *Dicraeus orientalis*, respectively (Figure 5). Interestingly, *E. tuberculifera* exhibits a greater genetic distance from the other *Elachiptera* species. Both the phylogenetic analyses showed that *E. decipiens* was closely related to *E. insignis.*

**Figure 4.**
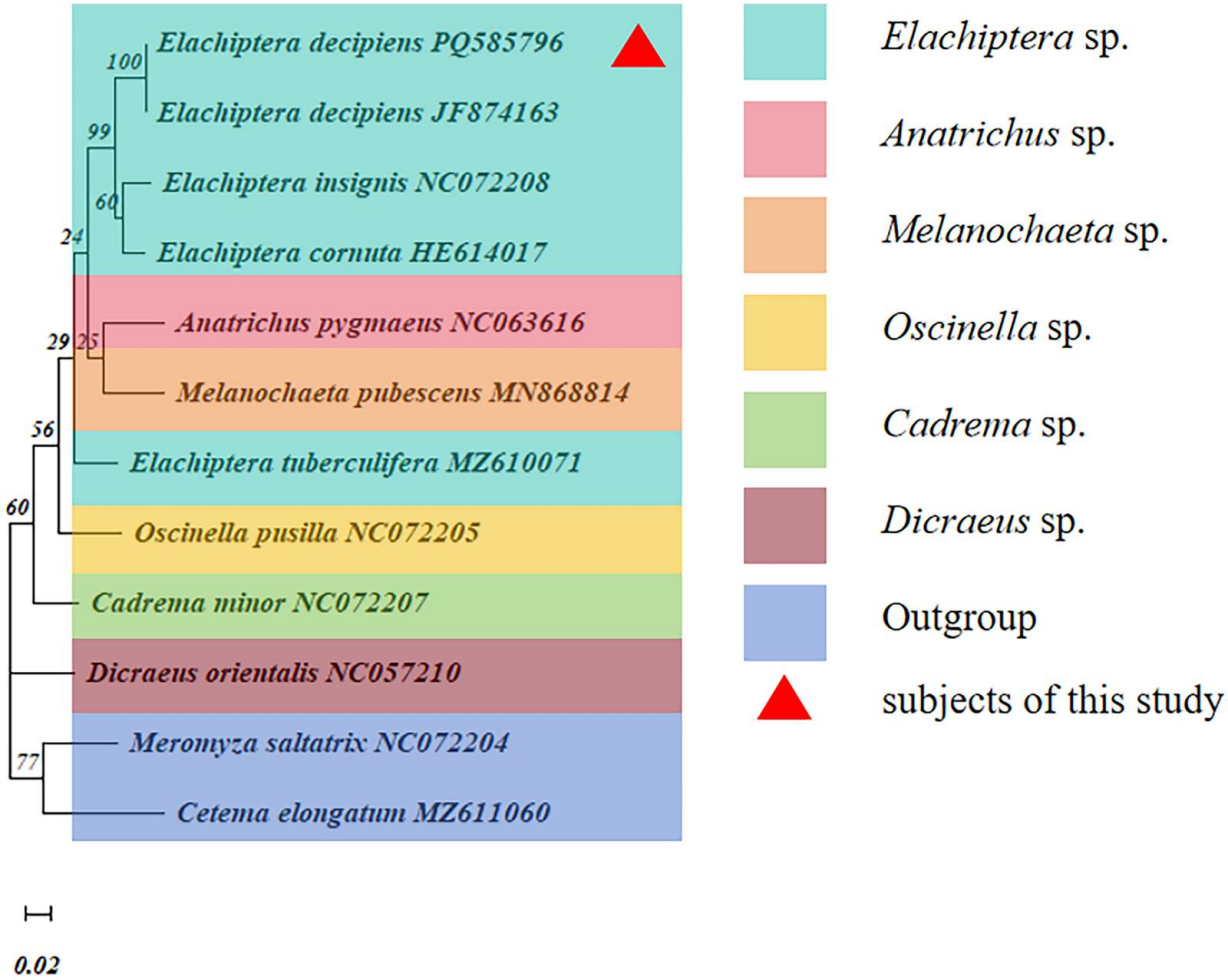
ML phylogenetic tree based on the COI gene. The larger the number on the branch, the higher the proof reliability. Alphanumeric terms indicate the accession number of the GenBank. The following sequences were used: *Elachiptera decipiens* PQ585796 (this study); *Elachiptera decipiens* JF874163 (unpublished); *Elachiptera insignis* NC 072208 (unpublished); *Elachiptera cornuta* HE614017 (unpublished); *Anatrichus pygmaeus* NC063616 (unpublished); *Melanochaeta pubescens* MN868814 (Ferreira et al. 2020); *Elachiptera tuberculifera* MZ610071 (unpublished); *Oscinella pusilla* NC072205 (Liu et al. 2024); *Cadrema minor* NC072207 (unpublished); *Dicraeus orientalis* NC057210 (unpublished); *Meromyza saltatrix* NC072204 (Liu et al. 2024); *Cetema elongatum* MZ611060 (unpublished).

## Discussion and conclusions

The mitochondrial genome size in Diptera insects varies from 11,339 bp to 19,517 bp (Zhang et al. 2013). In the present study, the complete mtDNA of *E. decipiens* was 18,344 bp in length, and the genomes exhibited a noticeable A + T bias (82.48%), which is consistent with the characteristics of insect mtDNA (Boore 1999) and Chloropidae species (Liu et al. 2024). Wang et al. (2021) reported that the complete mtDNA of *Chlorops oryzae* had an A + T value of 79.22%. Liu et al. (2024) reported that the A + T value of chloropid species ranged from 78.5% (*Thaumatomyia glabra*) to 80.9% (*Elachiptera insignis*). Compared with the two species, *C. oryzae* and *E. insignis*, *E. decipiens* has a slightly higher AT value. The sequencing results reveal that the *COI* promoter TCG of *E. decipiens* is the same as that of *E. insignis* determined by Liu et al. (2024).

Until now, the subfamily-level and genus-level relationships of Chloropidae have been a topic of debate. Mlynarek and Wheeler (2018) proposed that *Elachiptera* was closely related to *Melanochaeta* based on adult morphological characteristics including the cephalic bristling and the shape of the scutellum. Liu et al. (2024) suggested that *Elachiptera* was most closely related to *Anatrichus* based on the mitogenome sequences. In our studies, *E. decipiens* showed a close genetic relationship both to *Anatrichus pygmaeus* and *Melanochaeta pubescens*, which is in accordance with the previous research (Mlynarek and Wheeler 2018; Liu et al. 2024).

Regarding the lower bootstrap values observed in Figure 5, there are two primary reasons. First, the sequences are phylogenetically close and exhibit a high degree of similarity, which contributes to the lower bootstrap values. Second, during sequence alignment, we aligned and trimmed the sequences based on the shortest one, resulting in sequences approximately 300 bp in length, which is relatively short. The high similarity among the remaining parts further contributes to the lower bootstrap values. Additionally, due to data constraints, we are unable to obtain mitochondrial whole-genome data, as depicted in Figure 3, for analyzing the Oscinellinae subfamily in Figure 5. Consequently, our analysis of the Oscinellinae subfamily remains preliminary. Improved results are anticipated as more data become available in the future.

Mitogenome has been not only widely used for investigations into phylogeny, phylogeography, and population genetics (Cameron 2014; Lv et al. 2015; Du et al. 2019; Huang and Zhang 2020), but also to develop the rapid identifying method for invasive species, such as *Daktulosphaira vitifoliae* and *Trogoderma granarium* (Agarwal et al. 2020; Wu et al. 2023), and tissue of cattle origin (Kumari et al. 2019). *E. decipiens* is seen as an invasive pest in China, but difficulties remain in distinguishing this species from similar ones. The mitogenome sequence of *E. decipien*s would assist border ports and in fields in its effective rapid identification as well as pest control at the early stages of monitoring.

In conclusion, we sequenced mitochondrial genomes of *E. decipiens*, and suggested the phylogenetic relationships of the species with several other *Elachiptera* spp. This study may provide some valuable genetic resources to understand Oscinellinae subfamily evolution, and to develop rapid identification method for *E. decipiens*.

## Supplementary Material

Supplemental Material

## Data Availability

The genome sequence data that support the findings of this study are openly available in GenBank of NCBI at https://www.ncbi.nlm.nih.gov/ under the accession number PQ585796. Raw sequencing reads used here have been deposited in the SRA database of NCBI under accession number SRR31912344. The associated ‘BioProject’ and ‘Bio-Sample’ numbers are PRJNA1202719 and SAMN45963734, respectively.
