# Supplementary figures and images for: The complete mitochondrial genome analysis of Elachiptera decipiens (Loew, 1863) (Diptera: Chloropidae)
Source: Mitochondrial DNA B Resour. 2026 Apr 10;11(5):599–603. doi: 10.1080/23802359.2026.2652762 (PMC13072685; doi:10.1080/23802359.2026.2652762)

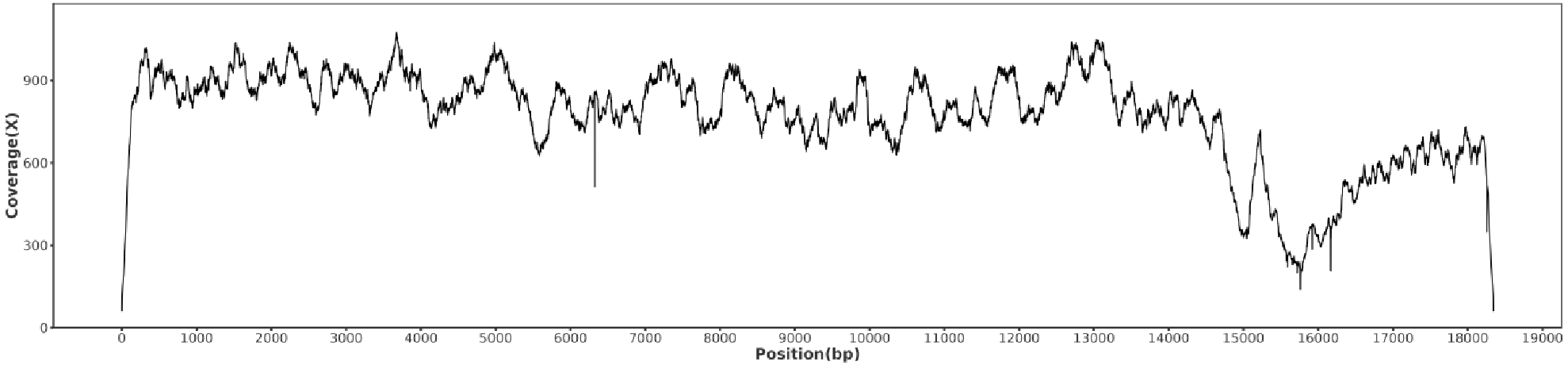

Supplement: Supplemental Material [file TMDN_A_2652762_SM5216.tif]
